# Effect of Modified Levopimaric Acid Diene Adducts on Mitochondrial and Liposome Membranes

**DOI:** 10.3390/membranes12090866

**Published:** 2022-09-08

**Authors:** Mikhail V. Dubinin, Vyacheslav A. Sharapov, Alena A. Semenova, Lyudmila V. Parfenova, Anna I. Ilzorkina, Ekaterina I. Khoroshavina, Natalia V. Belosludtseva, Sergey V. Gudkov, Konstantin N. Belosludtsev

**Affiliations:** 1 Department of Biochemistry, Cell Biology and Microbiology, Mari State University, pl. Lenina 1, 424001 Yoshkar-Ola, Russia; 2 Institute of Petrochemistry and Catalysis, Ufa Federal Research Center, Russian Academy of Sciences, Prospekt Oktyabrya 141, 450075 Ufa, Russia; 3 Institute of Theoretical and Experimental Biophysics, Russian Academy of Sciences, Institutskaya 3, 142290 Pushchino, Russia; 4 Prokhorov General Physics Institute of the Russian Academy of Sciences, Vavilov St. 38, 119991 Moscow, Russia

**Keywords:** levopimaric acid diene adducts, mitochondria, oxidative phosphorylation, ROS, liposomes, membrane permeability

## Abstract

This paper demonstrates the membranotropic effect of modified levopimaric acid diene adducts on liver mitochondria and lecithin liposomes. We found that the derivatives dose-dependently reduced the efficiency of oxidative phosphorylation of mitochondria due to inhibition of the activity of complexes III and IV of the respiratory chain and protonophore action. This was accompanied by a decrease in the membrane potential in the case of organelle energization both by glutamate/malate (complex I substrates) and succinate (complex II substrate). Compounds **1** and **2** reduced the generation of H_2_O_2_ by mitochondria, while compound **3** exhibited a pronounced antioxidant effect on glutamate/malate-driven respiration and, on the other hand, caused ROS overproduction when organelles are energized with succinate. All tested compounds exhibited surface-active properties, reducing the fluidity of mitochondrial membranes and contributing to nonspecific permeabilization of the lipid bilayer of mitochondrial membranes and swelling of the organelles. Modified levopimaric acid diene adducts also induced nonspecific permeabilization of unilamellar lecithin liposomes, which confirmed their membranotropic properties. We discuss the mechanisms of action of the tested compounds on the mitochondrial OXPHOS system and the state of the lipid bilayer of membranes, as well as the prospects for the use of new modified levopimaric acid diene adducts in medicine.

## 1. Introduction

Abietane-type tricyclic diterpenoids are a class of compounds resulting from the condensation of four isoprene units, providing a wide range of possible chemical structures. These compounds are of interest for various modifications due to a broad spectrum of potential biological activity of semi-synthetic analogs, which are of particular pharmacological and pharmaceutical interest. For example, antitumor [1], antibacterial [2], antiviral [3], antiparasitic [4], antiulcer [5], anti-inflammatory [6] and antioxidant [7] properties, apoptosis induction [8], and effects on gastrointestinal and hepatic disease [9] have been reported for these compounds. Dehydroabietylamine, abietic and dehydroabietic acids and their synthetic derivatives are promising anticancer agents with potent inhibitory activity against various types of human cancer cell lines, such as prostate, ovarian, breast, liver, lung and cervical cancer cells. Their mechanisms of action are varied and include induction of apoptosis, DNA binding, inhibition of tubulin polymerization, and disruption of intracellular cholesterol transport [10,11]. It is also known that some representatives of abietanes (for example, carnosic acid) prevent H_2_O_2_-triggered mitochondrial redox impairment and dysfunction (i.e., TCA impairment) and cell death in SH-SY5Y cells by a mechanism involving the transcription factor Nrf2 [12]. Another example, 2,14-dichlorodehydro-abietic acid, reduced Ca^2+^ overload in isolated mitochondria, activated mitoK_Ca_ channels in inside-out patches of mitochondrial membrane, facilitated flavoprotein oxidization in myocytes, and increased cellular viability under simulated ischemia [13]. Dehydroabietic acid derivatives conjugated with acyl-thiourea peptide moiety induce apoptosis in HeLa cells via the mitochondrial pathway, including an increase in the production of reactive oxygen species (ROS) and intracellular Ca^2+^, loss of mitochondrial membrane potential, and activation of caspase-3 [14]. Additionally, 15-oxospiramilactone is a diterpenoid derivative capable of inducing structural and functional conversion of the Bcl-2 family proteins that regulate the mitochondrial membrane permeability in apoptosis by a Bax/Bak-independent mechanism [15].

Levopimaric acid is an abietane-type diterpene that exhibits anti-cancer properties by inducing mitochondrial dysfunction in cancer cells under in vitro conditions [16]. Diene adducts of levopimaric acids and their modified derivatives have demonstrated antitumor effects as well. 1,4-Dihydroxyiminodihydroquinopimaric acid methyl ester showed in vitro cytotoxic activity against various human cancer cell lines, and in vivo tests for this compound showed antitumor activity against breast carcinoma and colon adenocarcinoma [17]. Cyanoethyl derivatives of dihydroquinopimaric acid showed a high antiproliferative effect in vitro against Jurkat, K562, U937, and HeLa tumor cell cultures and proved to be effective inducers of apoptosis with a dose-dependent effect on the S and G2 phases of the cell cycle [18].

**Figure 1:**
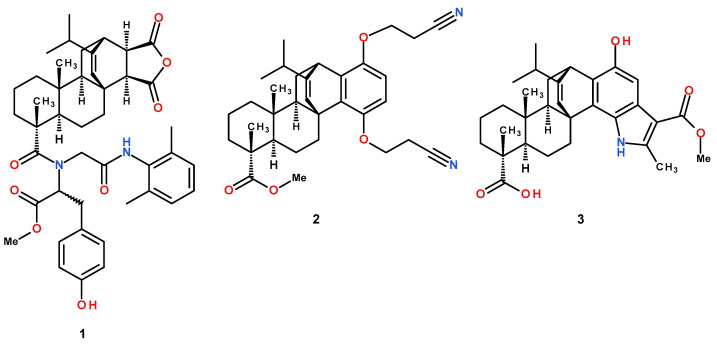
Structure of modified levopimaric acid diene adducts.

Given the important role of mitochondria in the effect of terpenoids, including abietanes, an important task is to identify their molecular targets. Therefore, in the present work, we studied the membranotropic effects of various types of modified levopimaric acid diene adducts (compounds **1**–**3**, Figure 1) on isolated rat liver mitochondria and estimated adduct-induced changes in major functional parameters of these organelles: (1) respiration and oxidative phosphorylation; (2) activity of complexes of the mitochondrial respiratory chain; (3) ROS production; (4) effects of compounds on the state and permeability of the inner mitochondrial membrane. Finally, we also used large unilamellar lecithin liposomes to clarify the effect of these agents on the lipid component of membranes. We revealed a dose-dependent decrease in the functional activity of mitochondria under the action of compounds, which manifested itself as a decrease in the efficiency of oxidative phosphorylation and a decrease in the membrane potential of organelles, which was also accompanied by a change in the generation of hydrogen peroxide. The tested agents were found to have a pronounced membranotropic effect on mitochondria with different efficiencies, inducing a decrease in the overall fluidity of the mitochondrial membranes and their permeabilization, and also had a protonophoric effect and suppressed the activity of mitochondrial respiratory chain complexes. In the case of lecithin liposomes, we also noted the ability of modified levopimaric acid diene adducts to induce nonspecific permeability of their membranes, which also confirms the membranotropic nature of these agents.

## 2. Materials and Methods

### 2.1. Reagents and Chemicals

Medium components, inorganic chemicals, and sulforhodamine B (SRB) were purchased from Sigma-Aldrich (St. Louis, MO, USA). Phosphatidylcholine (lecithin) was purchased from Avanti Polar Lipids (Avanti Polar Lipids Inc., Alabaster, AL, USA). The derivatives used in this work were prepared in ethanol as a 5 mM stock solution.

### 2.2. Synthesis of Modified Levopimaric Acid Diene Adducts

Compounds **1**–**3** (Figure 1) were synthesized according to published methods: methyl N-(2-((2,6-dimethylphenyl)amino)-2-oxoethyl)-N-(12-isopropyl-6,9a-dimethyl-1,3-dioxo-3,3a,4,5,5a,6,7,8,9,9a,9b,10,11,11a-tetradecahydro-1H-3b,11-ethenophenanthro [1,2-c]furan-6-carbonyl)tyrosinate (**1**) [19], methyl 1,4-bis(2-cyanoethoxy)-13-isopropyl-7,10a-dimethylhexadecahydro-1H-4b,12-etheno chrysene-7-carboxylate (**2**) [18] and 1-Hydroxy-14-isopropyl-3-(methoxycarbonyl)-4,8,11a-trimethyl-5,6,7,7a,8,9,10,11,11a,11b,12,13-dodecahydro-5c,13-ethenophenanthro-[2,1-g]indole-8-carboxylic acid (**3**) [20].

### 2.3. Isolation of Rat Liver Mitochondria

Mitochondria were isolated from the liver of 210–250 g male Wistar rats using a convenient technique of differential centrifugation [21]. The isolation medium contained 250 mM sucrose, 1 mM EGTA, and 5 mM Hepes/KOH buffer (pH 7.4). The mitochondrial protein concentration was determined by the biuret method with BSA used as standard.

### 2.4. Determination of Mitochondrial Respiration and Oxidative Phosphorylation

The rate of oxygen consumption was measured polarographically with a Clarke type oxygen electrode and an Oxygraph Plus device (Hansatech Instruments, Norfolk, UK) under continuous stirring at 25 °C [22]. The medium contained 200 mM sucrose, 20 mM KCl, 0.5 mM EGTA, 5 mM KH_2_PO_4_, 5 mM potassium succinate, 1 μM rotenone and 10 mM Hepes/KOH, pH 7.4. Estimated were the mitochondrial respiration in resting state (i.e., basal mitochondrial respiration in the presence of exogenous substrates or state 2), in state 3 (exogenous substrates plus ADP), in state 4 (after ADP exhaustion) and in uncoupled state 3U_DNP_ in the presence of an uncoupler (50 μM 2,4-dinitrophenol) [22]. The rates of substrate oxidation were expressed as nmol O_2_ × min^−1^ × mg^−1^ mitochondrial protein. Respiratory control ratio (RC = state 3/state 4), and *ADP/O* ratio were determined according to [23]. The concentration of mitochondrial protein was 1 mg/mL.

### 2.5. Measuring Activity of Complexes of the Mitochondrial Electron Transport Chain (ETC)

The effect of derivative on the activity of mitochondrial electron transport chain complexes (I, II, III, IV) was estimated using specific redox reactions according to the protocol using a Multiskan GO plate reader (Thermo Fisher Scientific, Waltham, MA, USA) [23]. The concentration of mitochondrial protein was about 50 µg/mL.

### 2.6. Monitoring of Mitochondrial Membrane Potential

The mitochondrial Δψ was evaluated with the fluorescent probe safranine O (λ_ex_ = 520 nm; λ_em_ = 580 nm) using a Varioskan LUX plate spectrofluorometer (Thermo Fisher Scientific, Waltham, MA, USA) [24]. Rat liver mitochondria (0.5 mg/mL) were incubated in a medium containing 210 mM mannitol, 70 mM sucrose, 10 μM EGTA, 10 µM safranine O, and 10 mM Hepes/KOH buffer, pH 7.4. A mixture of 2.5 mM glutamate plus 2.5 mM malate or 5 mM succinate were used as respiratory substrates. In the latter case, the incubation medium was supplemented with 1 μM rotenone.

### 2.7. Production of H_2_O_2_ by Rat Liver Mitochondria

Mitochondrial H_2_O_2_ generation was measured using an Amplex Red fluorescent indicator (λ_ex_ = 560 nm; λ_em_ = 590 nm) and plate reader [24]. Liver mitochondria (0.15 mg protein per mL) were incubated at 37 °C in the following buffer: 210 mM mannitol, 70 mM sucrose, 1 a.u./mL horseradish peroxidase, 10 μM Amplex Red, 10 mM EGTA and 10 mM Hepes/KOH (pH 7.4). The amount of H_2_O_2_ generated was calculated using a calibration curve. For this purpose, on the day of the experiment, a fresh solution of hydrogen peroxide was prepared, and its concentration was determined by the molar absorption coefficient E_240_ = 43.6 M^−1^ × cm^−1^.

### 2.8. Monitoring Optical Density of Mitochondrial Suspension

Mitochondrial swelling was assessed at 540 nm using a plate reader. The following buffer was used: 210 mM mannitol, 70 mM sucrose, 5 mM succinic acid, 2 mM rotenone, 10 mM EGTA and 10 mM Hepes/KOH (pH 7.4). The concentration of mitochondrial protein was 0.5 mg/mL.

The protonophore effect of the test preparations was assessed by low-amplitude swelling of mitochondria incubated in isotonic ammonium nitrate solution at 600, as shown previously [25]. For this purpose, non-energized mitochondria (0.5 mg protein per mL) were incubated at room temperature (~22 °C) in a buffer of the following composition: 135 mM NH_4_NO_3_, 0.1 mM EGTA, and 10 mM Hepes/KOH (pH 7.4).

### 2.9. Evaluation of Mitochondrial Membrane Fluidity by Generalized Polarization (GP) of Laurdan

Fluidity of the mitochondrial membranes was estimated by measuring the fluorescence of the environment-sensitive fluorescent probe laurdan [26,27] in 96-well plates. For this purpose, liver mitochondria (0.5 mg protein per mL) were incubated at 37 °C in a buffer of the following composition: 210 mM mannitol, 70 mM sucrose, 5 mM succinic acid, 2 μM rotenone, 10 μM EGTA, 1 μM laurdan and 10 mM Hepes/KOH (pH 7.4). The probe fluorescence was initiated at 340 nm, and the emission was evaluated at 430 and 490 nm using plate spectrofluorometer. The generalized polarization (GP) parameter was calculated as (I_430_ − I_490_)/(I_430_ + I_490_) where I is the emission intensity at corresponding wavelengths [26,27].

### 2.10. Construction of Liposomes

The extrusion method was used to obtain liposomes (large unilamellar vesicles or LUV). For this purpose, 7.5 mg of dry lecithin was incubated for several hours in 0.75 mL of the following buffer: 10 mM Tris/HCl (pH 8.5), 50 mM SRB and 50 μM EGTA. Five freezing/thawing cycles were performed before the multilamellar liposome suspension was extruded 11 times through a 0.1 μm polycarbonate membrane using a microextruder (Avanti Polar Lipids, Birmingham, AL, USA). After extrusion, liposomes were applied on a Sephadex G-50 column to remove the external SRB. The release of SRB from liposomes incubated in 10 mM Tris/HCl (pH 7.5), 50 μM EGTA, and 40 mM KCl buffer was evaluated by the increase in SRB fluorescence using an Ocean Optics FLAME-T-UV-VIS fiber-optic system (Ocean Optics Inc., Dunedin, USA), as described earlier [21]. A 0.1% solution of Triton X-100 was added to release all of the loaded SRB.

### 2.11. Statistical Analysis

The data were analyzed using GraphPad Prism 8 and Excel software and were presented as means ± SEM. The statistical significance of the differences between the means was evaluated using one-way analysis of variance (ANOVA) followed by the Tukey multiple comparison post hoc test, and *p* < 0.05 was selected as an indicator of statistical significance.

## 3. Results

### 3.1. Modified Levopimaric Acid Diene Adducts Reduce the Efficiency of Oxidative Phosphorylation in Rat Liver Mitochondria

In the first part of the work, we studied the effect of compounds on one of the main functions of mitochondria, respiration and oxidative synthesis of ATP. To do this, we evaluated the rate of mitochondrial respiration with glutamate/malate (substrates of complex I of the respiratory chain) or succinate (a substrate of complex II) plus rotenone in the presence and absence of the studied agents. Table 1 shows that compound **1** had no effect on mitochondrial respiration. At the same time, compounds **2** and **3** dose-dependently reduced the rate of glutamate/malate-dependent respiration of organelles in the phosphorylating state (state 3), and at a concentration of 20 μM, this effect was statistically significant. We also noted a 1.4-fold decrease in the maximal rate of organelle respiration induced by protonophore uncoupler DNP in the presence of 20 μM compound **3**. On the other hand, compounds **2** and **3** increased the rate of respiration in state 4, the latter being also the most effective and causing a 1.4-fold stimulation of respiration. Moreover, 20 μM compound **3** stimulated organelle respiration in state 2 as well (Table 1). As a result, we noted a significant decrease in the respiratory control ratio of glutamate/malate-fueled liver mitochondria, most pronounced in the case of compound **3** (1.9-fold decrease). Moreover, compound **3** already at a concentration of 10 μM also reduced the *ADP/O* ratio reflecting the suppression of ATP synthesis by mitochondria.

In the case of succinate-driven mitochondrial respiration, we also noted a dose-dependent suppression of the respiration rate in state 3 in the presence of all tested agents (Table 2); in this case, compound **2** was slightly more effective and caused a 1.4-fold decrease in the oxygen consumption rate. The rate of DNP-induced respiration also decreased dose-dependently in the presence of compounds **2** and **3** (1.2-fold). We also noted the stimulating effect of 20 μM of compounds **2** and **3** on the respiration of organelles in state 4, inducing a 1.2- and 1.4-fold increase in the rate of oxygen consumption, respectively. All of these events were also accompanied by a dose-dependent decrease in the respiratory control ratio; in this case, compounds **2** and **3** were approximately equally effective and caused a 1.8-fold decrease in this parameter.

**Figure 2:**
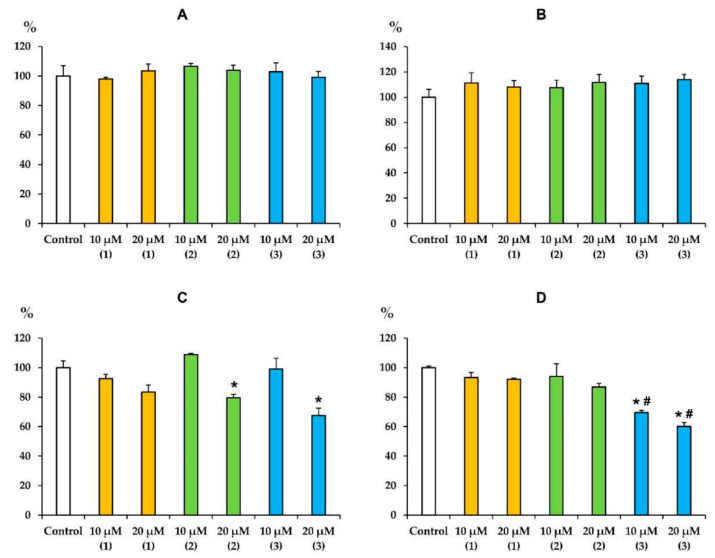
Effects of modified levopimaric acid diene adducts on the activity of complexes of the mitochondrial respiratory chain (values in % of activity compared with control). In the absence of conjugate (control), the activities of complexes I (panel **A**), II (panel **B**), III (panel **C**) and IV (panel **D**) were 365 ± 11, 412 ± 12, 679 ± 9 and 477 ± 4 nmol/min/mg protein, respectively. The activity values in the absence of conjugate were taken as 100%. The results are presented as means ± SEM (*n* = 4). * *p* < 0.05 (versus control), # *p* < 0.05 (versus corresponding concentrations of compounds **1** and **2**).

We have previously shown that terpenoids and, in particular, triterpenoids and their various derivatives, are able to accumulate in the inner mitochondrial membrane and significantly affect the activity of the complexes of the mitochondrial respiratory chain [25,28,29,30]. Given the pronounced inhibitory effect of modified levopimaric acid diene adducts on mitochondrial respiration in state 3 and 3U_DNP_ (Table 1 and Table 2), the same effects should be expected. Figure 2 shows that all tested compounds did not affect the activity of complexes I and II. At the same time, compounds **2** and **3** at a concentration of 20 μM significantly suppressed the activity of complex III, while compound **1** only demonstrated a similar trend. Compound **3** also suppressed the activity of cytochrome *c* oxidase (complex IV), and a statistically significant effect was found already at 10 μM of this agent (Figure 2D). These data are generally consistent with the results obtained by measuring mitochondrial respiration (Table 1 and Table 2).

**Figure 3:**
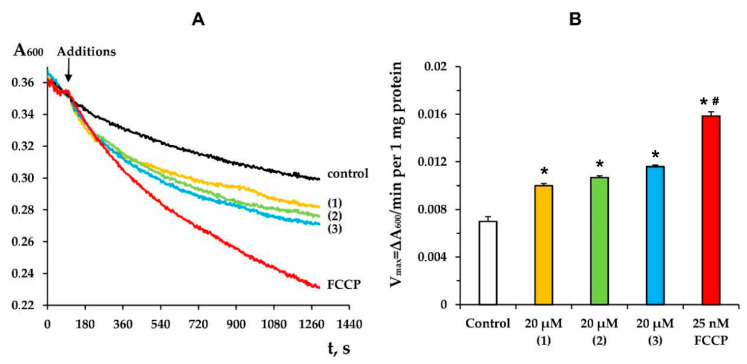
The kinetics (**A**) and rate (**B**) of mitochondrial swelling in NH_4_NO_3_ buffer induced by 20 µM of tested compounds or 25 nM FCCP. Control curve obtained in the absence of additions. Panel A shows typical curves recorded with the same sample during the same experiment. Similar results were observed in three other independent experiments. (**B**) shows means ± SEM (*n* = 4). * *p* < 0.05 (versus control), # *p* < 0.05 (versus tested compounds).

In turn, the stimulation of mitochondrial respiration in state 4 can be explained by the known ability of terpenoids and their derivatives to transport protons through the inner mitochondrial membrane, exerting a protonophore effect on mitochondria [25,28,30]. In this work, we also evaluated the effect of modified levopimaric acid diene adducts on mitochondrial swelling in isotonic NH_4_NO_3_ medium. It is known that the inner mitochondrial membrane is permeable to NO_3_^−^ and NH_3_, but not to H^+^ and NH_4_^+^; therefore, mitochondria show low-rate swelling in an isosmotic NH_4_NO_3_ solution [25,31]. However, an increase in the permeability of the mitochondrial membrane for H^+^ under the condition of simultaneous passive diffusion of NH_3_ promotes the accumulation of osmotically active NH_4_NO_3_ in the matrix and leads to swelling of mitochondria. This was observed when the known protonophore uncoupler carbonyl cyanide 4-(trifluoromethoxy) phenylhydrazone (FCCP) was added to deenergized mitochondria (Figure 3). In this case, all three studied modified levopimaric acid diene adducts increased the rate of mitochondrial swelling (Figure 3B). In this regard, it can be assumed that the increase in the rate of respiration of organelles in state 4, induced by the tested agents, may be due to their protonophore effect.

**Figure 4:**
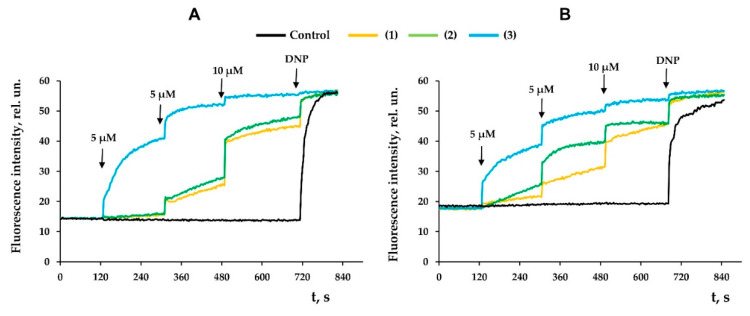
Effects of modified levopimaric acid diene adducts on membrane potential of rat liver mitochondria fueled by glutamate/malate (**A**) or succinate (**B**). Black curve (control) obtained in the absence of tested compounds. DNP (50 μM) was added to induce maximum decrease in membrane potential. The picture shows typical curves recorded with the same sample during the same experiment. Similar results were observed in two other independent experiments.

Suppression of the activity of respiratory chain complexes, as well as an increase in the proton permeability of the inner mitochondrial membrane, are known to be accompanied by a decrease in the membrane potential of organelles. Indeed, Figure 4 shows that all tested compounds dose-dependently reduced the membrane potential (Δψ) of rat liver mitochondria, while the effectiveness of the compounds increased from compound **1** to compound **3**, which is also consistent with the data obtained from the assessment of organelle respiration (Table 1 and Table 2).

### 3.2. Modified Levopimaric Acid Diene Adducts Modulate Hydrogen Peroxide Production by Liver Mitochondria

**Figure 5:**
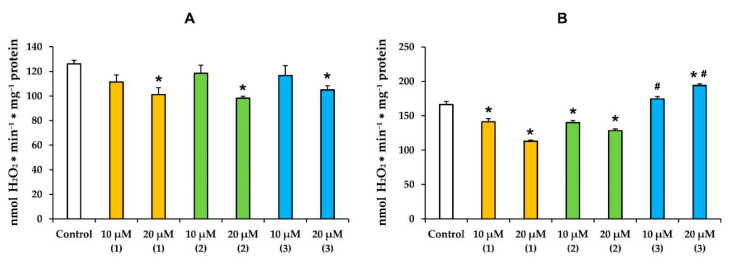
The rate of H_2_O_2_ generation by rat liver mitochondria fueled by glutamate/malate (**A**) or succinate (**B**) in the presence of modified levopimaric acid diene adducts. The results are presented as means ± SEM (*n* = 4). * *p* < 0.05 (versus control), # *p* < 0.05 (versus corresponding concentrations of compounds **1** and **2**).

It is known that the mitochondria-targeted action of terpenes is accompanied by overproduction of ROS [25,28,29,30,31,32,33]. In the present work, we also evaluated the effect of modified levopimaric acid diene adducts on the rate of H_2_O_2_ production by rat liver mitochondria (Figure 5). One can see that in the case of glutamate/malate-fueled respiration, all tested compounds induced a dose-dependent decrease in mitochondrial hydrogen peroxide production with the same efficiency (1.2-fold) (Figure 5A). Compounds **1** and **2** showed the same effect in the case of mitochondria energized with succinate, and at a concentration of 20 μM, they reduced H_2_O_2_ production by 1.5 and 1.4 times, respectively (Figure 5B). At the same time, compound **3**, on the contrary, caused overproduction of hydrogen peroxide in succinate-fueled mitochondria, increasing its generation by almost 1.2 times.

### 3.3. Modified Levopimaric Acid Diene Adducts Decrease the Overall Fluidity of the Mitochondrial Membranes and Induce Permeabilization of Mitochondrial Membranes as well as Lecithin Liposome Membranes

We have previously shown that terpenoids and their derivatives, being membranotropic agents, can not only modify the work of mitochondrial electron carriers, influencing the functioning of organelles, but also affect the overall fluidity and permeability of the lipid bilayer of membranes, which was also demonstrated on artificial liposome membranes [28]. Given the common structure and hydrophobic nature, we can assume a similar effect for the modified levopimaric acid diene adducts tested in this work.

**Figure 6:**
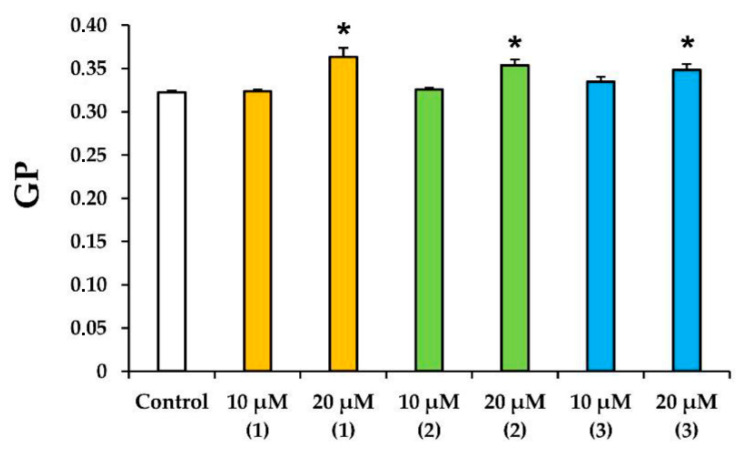
Effects of modified levopimaric acid diene adducts on laurdan GP in mitochondria. The results are presented as means ± SEM (*n* = 4). * *p* < 0.05 (versus control).

The effect of the tested preparations on the state of rat liver mitochondrial membranes was assessed using a laurdan fluorescent probe. The generalized polarization (GP) of this probe was the relative difference between the fluorescence intensities at two wavelengths (red and blue peaks) and reflected the hydration of the membrane lipid bilayer and the mobility of water molecules in the region of the lipid heads [26,27]. One can see that all tested compounds at a concentration of 20 µM caused a statistically significant increase in the laurdan GP (Figure 6). In other words, modified levopimaric acid diene adducts reduced the mobility of water molecules near lipid heads and increased the microviscosity of the mitochondrial membranes.

**Figure 7:**
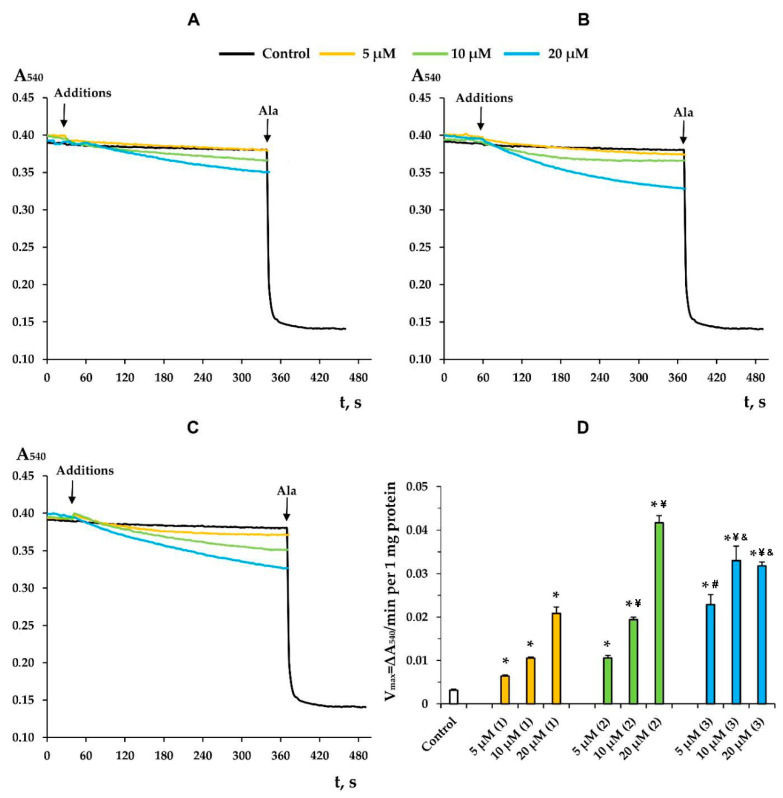
The swelling of liver mitochondria induced by modified levopimaric acid diene adducts: compound **1** (**A**), compound **2** (**B**) and compound **3** (**C**). Control curves obtained in the absence of additions. Alamethicin (Ala) (5 μg/mL) was added to induce maximal swelling. The picture shows typical curves recorded with the same sample during the same experiment. Similar results were observed in three other independent experiments. Panel **D** shows the rates of swelling induced by various concentrations of tested agents. The results are presented as means ± SEM (*n* = 4). * *p* < 0.05 (versus control), # *p* < 0.05 (versus 5 μM of compounds **1** and **2**), ¥ *p* < 0.05 (versus corresponding concentration of compound **1**), & *p* < 0.05 (versus corresponding concentration of compound **2**).

**Figure 8:**
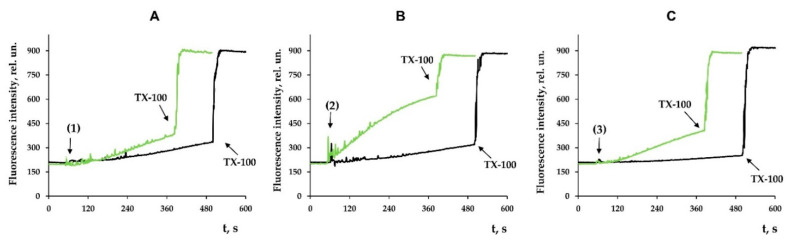
Effects of modified levopimaric acid diene adducts on SRB release from lecithin liposomes. The incubation medium contained 10 mM Tris/HCl (pH 7.5), 50 μM EGTA, and 40 mM KCl. Additions: 10 μM (*black lines*) and 30 μM (*green lines*) of tested compounds **1** (**A**), **2** (**B**) and **3** (**C**). Other additions: 0.1% Triton X-100. The picture shows typical curves recorded with the same sample during the same experiment. Similar results were observed in two other independent experiments.

Along with this, terpenoids, being surface-active substances, are able to induce nonspecific permeabilization of the lipid bilayer of membranes [25,28,29,30]. Indeed, one can see that the tested compounds caused a dose-dependent decrease in the optical density of the mitochondrial suspension (Figure 7). This indicated the high-amplitude swelling of the organelles due to the formation of the nonspecific permeability of the inner membrane. Compounds **2** and **3** were the most effective, as the rate of swelling of mitochondria was the highest in their presence (Figure 7D). Interestingly, this process was also insensitive to cyclosporin A (CsA), an inhibitor of the mitochondrial permeability transition pore (data not shown). This is typical of terpenoids and may reflect that modified levopimaric acid diene adducts induce CsA-insensitive lipid bilayer permeabilization and swelling of organelles. These results were also confirmed in experiments on artificial lipid systems—unilamellar lecithin liposomes. Figure 8 shows that all tested compounds induced a dose-dependent release of the sulforodamine B (SRB) fluorescent probe from liposomes, indicating nonspecific permeability of the lipid bilayer of the vesicles. In this case, compound **2** exhibited the highest activity, which corresponded to the data obtained by assessing the permeability of mitochondrial membranes (Figure 7).

## 4. Discussion

Levopimaric acid is an abietane-type of diterpene resin acid and the major constituent of pine oleoresin. It shows various biological activities including antioxidant, antibacterial and cardiovascular effects [34]. The mitochondria-targeted effects of this acid have also been described and can be used to combat tumor cells. Semi-synthetic analogues of levopimaric acid have been obtained, exhibiting a wide range of therapeutic effects [35,36,37,38]. Considering that mitochondria can be the target of levopimaric acid and its derivatives, we studied the effect of modified levopimaric acid diene adducts on a number of major parameters reflecting the functional activity of rat liver mitochondria. We found that all derivatives dose-dependently affected mitochondrial respiration and oxidative phosphorylation. Here, two oppositely directed effects may be distinguished. On the one hand, there was a decrease in the rate of oxygen consumption in the phosphorylating state (state 3) associated with ATP synthesis (Table 1 and Table 2). In this case, compound **1** exhibited this effect only when organelles were energized with succinate (substrate of complex II), while compounds **2** and **3** were also effective in the case of glutamate/malate-driven respiration of organelles initiated through complex I of the respiratory chain. One could assume that such an action of the tested agents is due to suppression of the activity of mitochondrial respiratory chain complexes. In particular, compounds **2** and **3** significantly suppressed the activity of complex III, and the effect of the latter was also pronounced in the case of cytochrome *c* oxidase (Figure 2). All of these events may lead to a decrease in the efficiency of electron transport to oxygen and the generation of proton motive force (Δ*p*) by these complexes. The same effects seem to be responsible for the decrease in the maximal rate of respiration induced by the protonophore uncoupler DNP. At the same time, compound **1** showed only a tendency to inhibit the activity of complex III, which may account for its weak effect on mitochondrial respiration. On the other hand, we noted the ability of compounds **2** and **3** to conversely increase the rate of mitochondrial respiration in state 4, which did not depend on the respiration substrate used (Table 1 and Table 2). This action of agents is presumably due to their ability to increase the permeability of the inner mitochondrial membrane for protons, in other words, by protonophore action (Figure 3). One way or another, all of these phenomena contribute to a significant decrease in the respiratory control ratio reflecting the efficiency of electron transport along the respiratory chain of mitochondria and the generation of Δ*p*. This was especially pronounced in the case of compound **3** and was also accompanied by a decrease in the *ADP/O* ratio in the case of energization of organelles by glutamate and malate (Table 1), which may also indicate the effect of this compound on the activity of ATP synthase. It is not surprising that the general inhibition of the functional activity of mitochondria by the tested agents was also accompanied by a corresponding decrease in the membrane potential of the organelles, which was most pronounced in the case of compound **3** showing activity already at a concentration of 5 µM (Figure 4).

Mitochondria and their electron transport chain are the main producers of ROS in living cells [39]. The intensity of ROS generation in mitochondria depends on many factors and is also modulated by a wide range of chemical agents. We found that the mitochondria-targeted action of modified levopimaric acid diene adducts was also accompanied by a change in the production of hydrogen peroxide by rat liver mitochondria. We noted the predominant dose-dependent antioxidant effect of the tested agents, leading to a decrease in H_2_O_2_ generation (Figure 5). This may be due to the protonophore effect of the compounds (Figure 3), leading to a decrease in the membrane potential of mitochondria, which, as is known, is accompanied by a decrease in the generation of ROS by respiratory chain complexes [39]. On the other hand, we noted an increase in hydrogen peroxide production by succinate-fueled mitochondria in the presence of compound **3** (Figure 3B), which seems to be due to inhibition of the activity of complex III, one of the main ROS producers in mitochondria [39], and significant suppression of complex IV, whose influence on ROS production also cannot be excluded (Figure 2).

It should be noted that modified levopimaric acid diene adducts also have a direct effect on the state of mitochondrial lipid membranes, reducing membrane fluidity, as evidenced by experiments with laurdan (Figure 6). This indicates the ability of the tested agents to accumulate in mitochondrial membranes and influence the activity of proteins localized in them, including ETC proteins, or to influence protein–lipid interactions, primarily associated with cardiolipin, an important structural component of the inner mitochondrial membrane supporting the correct assembly and operation of the ETC complexes [40]. Along with this, the compounds can directly affect the lipid bilayer of organelle membranes, inducing its nonspecific permeabilization, leading to mitochondrial swelling in mannitol/sucrose-based medium (Figure 7). In this case, compounds **2** and **3** were most effective. We noted that the permeability induced by the compounds was of a lipid nature, since it was not blocked by an inhibitor of the proteinaceous MPT pore cyclosporin A (data not shown). Moreover, this phenomenon was also observed in the case of artificial liposomal membranes and was accompanied by the release of the fluorescent dye SRB (Figure 8), which has a significant molecular weight (559 Da). However, due to differences in lipid composition (primarily the absence of cardiolipin) and the absence of proteins in liposomes, it is not yet possible to definitively extrapolate these results to mitochondrial membranes. First of all, we are talking about the differences in the concentration dependence of the effects on these two systems. Nevertheless, the results obtained on mitochondria and liposomes indicated the fundamental ability of the tested compounds to induce non-specific permeability of the lipid bilayer of membranes. This property of the tested compounds may be used to target regulation of mitochondrial permeability involved in cell death.

At this stage, we cannot exclude other mechanisms of action of the tested compounds that are not related to the impact of the compounds on the organization of the mitochondrial membrane lipids. In particular, the nitrile groups in compound **2** may function as nucleophile, and this may lead to nucleophilic attachment to NH fragments in proteins via hydrogen bonding interaction [41]. This may have an additional effect on the activity of mitochondrial and other proteins and requires further study.

The data obtained indicate that modified levopimaric acid diene adducts, depending on the structure, are able to exert a mitochondrial targeted effect with different efficiency, reducing the functional activity of organelles and inducing nonspecific permeability of their membranes. This can be used for the further development of promising therapeutic compounds capable of modulating the functioning of the bioenergetic apparatus of the cell in a targeted manner.

## Figures and Tables

**Table 1:** Effects of modified levopimaric acid diene adducts on the respiration of rat liver mitochondria fueled by glutamate and malate.

Additions	State 2	State 3	State 4	State 3U_DNP_	RC	*ADP/O*
	nmol O_2_ * min^−1^ * mg^−1^ Protein				rel. un.	
Control	2.50 ± 0.22	21.46 ± 0.55	3.18 ± 0.04	22.59 ± 1.75	6.75 ± 0.19	2.93 ± 0.03
Compound 1						
10 μM	2.42 ± 0.22	20.78 ± 0.58	2.99 ± 0.14	23.13 ± 0.97	6.97 ± 0.16	2.91 ± 0.05
20 μM	2.58 ± 0.21	19.80 ± 0.48	3.13 ± 0.10	20.58 ± 0.26	6.28 ± 0.03	2.90 ± 0.03
Compound 2						
10 μM	2.59 ± 0.18	20.14 ± 0.84	3.11 ± 0.20	24.02 ± 1.09	6.49 ± 0.15	2.94 ± 0.06
20 μM	3.25 ± 0.31	18.15 ± 0.17 *	3.77 ± 0.09 *	24.05 ± 0.35	4.82 ± 0.04 *	2.98 ± 0.02
Compound 3						
10 μM	3.16 ± 0.23	19.28 ± 0.13	3.75 ± 0.11 *	18.83 ± 0.23	5.15 ± 0.12 *	2.68 ± 0.03 *
20 μM	3.56 ± 0.12 *	16.19 ± 0.47 *	4.61 ± 0.08 *#	15.75 ± 0.68 *	3.51 ± 0.04 *#	2.60 ± 0.01 *

Oxygen consumption by mitochondria was driven by glutamate and malate. Table shows means ± SEM (*n* = 4). * *p* < 0.05 (versus control); # *p* < 0.05 (versus 20 μM compound **2**).

**Table 2:** Effects of modified levopimaric acid diene adducts on the succinate-fueled respiration of rat liver mitochondria.

Additions	State 2	State 3	State 4	State 3U_DNP_	RC	*ADP/O*
	nmol O_2_ * min^−1^ * mg^−1^ protein				rel. un.	
Control	7.78 ± 0.18	47.77 ± 0.25	8.65 ± 0.19	57.48 ± 0.52	5.53 ± 0.09	1.79 ± 0.05
Compound 1						
10 μM	7.01 ± 0.23	43.45 ± 0.35 *	7.83 ± 0.13	53.51 ± 1.00	5.55 ± 0.05	1.71 ± 0.05
20 μM	8.22 ± 0.19	38.54 ± 0.16 *	8.84 ± 0.11	53.39 ± 0.87	4.36 ± 0.04 *	1.75 ± 0.07
Compound 2						
10 μM	7.21 ± 0.50	42.32 ± 0.23 *	8.29 ± 0.15	52.51 ± 0.51	5.11 ± 0.12 *	1.83 ± 0.11
20 μM	7.94 ± 0.10	33.92 ± 0.98 *#	10.75 ± 0.49 *	46.11 ± 2.23 *	3.16 ± 0.06 *#	1.89 ± 0.06
Compound 3						
10 μM	7.48 ± 0.32	41.83 ± 1.32 *	8.64 ± 0.53	52.29 ± 2.11	4.85 ± 0.15 *	1.91 ± 0.08
20 μM	9.42 ± 0.27 *	37.53 ± 1.59 *	12.16 ± 0.26 *	47.73 ± 2.05 *	3.09 ± 0.07 *#	1.78 ± 0.01

Oxygen consumption by mitochondria was driven by succinate in the presence of rotenone. Table shows means ± SEM (*n* = 4). * *p* < 0.05 (versus control); # *p* < 0.05 (versus 20 μM compound **1**).

## Data Availability

The data presented in this study are available upon request from the corresponding author.
